# Pharmacological Treatments for Disordered Gambling: A Meta-analysis

**DOI:** 10.1007/s10899-018-09815-y

**Published:** 2018-12-20

**Authors:** Martina Goslar, Max Leibetseder, Hannah M. Muench, Stefan G. Hofmann, Anton-Rupert Laireiter

**Affiliations:** 1 Department of Psychology, University of Salzburg, Hellbrunnerstrasse 34, 5020 Salzburg, Austria; 2 Department of Clinical Psychology and Psychotherapy, Eberhard Karls University of Tuebingen, Schleichstraße 4, 72076 Tuebingen, Germany; 3 Department of Psychological and Brain Sciences, Boston University, 900 Commonwealth Avenue, 2nd Fl., Boston, MA 02215 USA; 4 Faculty of Psychology, University of Vienna, Liebiggasse 5, 1010 Vienna, Austria

**Keywords:** Gambling disorder, Pharmacological treatment, Meta-analysis

## Abstract

Disordered gambling is a public health concern associated with detrimental consequences for affected individuals and social costs. Currently, opioid antagonists are considered the first-line treatments to reduce symptoms of uncontrolled gambling. Only recently, glutamatergic agents and combined pharmacological and psychological treatments have been examined appearing promising options for the management of gambling disorder. A multilevel literature search yielded 34 studies including open-label and placebo-controlled trials totaling 1340 participants to provide a comprehensive evaluation of the short- and long-term efficacies of pharmacological and combined treatments. Pharmacological treatments were associated with large and medium pre-post reductions in global severity, frequency, and financial loss (Hedges’s *g*: 1.35, 1.22, 0.80, respectively). The controlled effect sizes for the outcome variables were significantly smaller (Hedges’s *g*: 0.41, 0.11, 0.22), but robust for the reduction of global severity at short-term. In general, medication classes yielded comparable effect sizes independent of predictors of treatment outcome. Of the placebo controlled studies, results showed that opioid antagonists and mood stabilizers, particularly the glutamatergic agent topiramate combined with a cognitive intervention and lithium for gamblers with bipolar disorders demonstrated promising results. However, more rigorously designed, large-scale randomized controlled trials with extended placebo lead-in periods are necessary. Moreover, future studies need to monitor concurrent psychosocial treatments, the type of comorbidity, use equivalent measurement tools, include outcome variables according to the Banff, Alberta Consensus, and provide follow-up data in order to broaden the knowledge about the efficacy of pharmacological treatments for this disabling condition.

Disordered gambling is defined based on the criteria for substance use including preoccupation with maladaptive behaviors, lack of control, tolerance, withdrawal, and continued behavior despite negative consequences (DSM 5; American Psychiatric Association 2013). Prevalence rates up to 5.8% worldwide (Calado and Griffiths 2016) indicate that problematic gambling is a public health concern associated with detrimental consequences for affected individuals in major areas of life (e.g., Raylu and Oei 2002) and social costs (e.g., Shaffer and Kidman 2004).

In accordance with the different phenomenological perspectives initially conceptualizing gambling pathology as obsessive–compulsive spectrum disorder and finally as behavioral addiction, various medication classes have been investigated over the years (for reviews see Grant et al. 2014c; Lupi et al. 2014). In the light of similarities between uncontrolled gambling and substance use disorders (e.g., Rash et al. 2016), clinical examinations focused on opioid antagonists currently appearing most likely to reduce symptoms of disordered gambling (e.g., Bartley and Bloch 2013). Research on comorbidity (e.g., Dell’Osso et al. 2005) and genetic aspects such as family history of alcohol use disorder (e.g., Grant et al. 2008b) further stimulated the exploration of mood stabilizers, glutamatergic agents, and combined pharmacological and psychological treatments which seem promising for the management of gambling disorder (De Brito et al. 2017; Kovanen et al. 2016; Pettorruso et al. 2014).

Although a number of systematic reviews have be conducted (e.g., Bullock and Potenza 2012), only three publications used meta-analytic strategies (Bartley and Bloch 2013; Leibetseder et al. 2011; Pallesen et al. 2007). The latest meta-analysis (Bartley and Bloch 2013) was limited to placebo controlled trials, and to the single outcome variable “gambling severity”. Furthermore, the impact of study quality and other moderators on treatment outcomes was not examined. Consequently, a comprehensive investigation of pharmacological treatment options for disordered gambling is still pending.

The primary objective of the present meta-analysis was to investigate the efficacy of pharmacological treatments for disordered gambling for reducing the (a) global severity, (b) frequency, and (c) financial loss from gambling after treatment (short-term effects) and at the latest follow-up (long-term effects). Based on the latest state of research, we expected (1) mood stabilizers and glutamatergic medications to be equally effective as opioid antagonists (Pettorruso et al. 2014) and (2) combined pharmacological and psychological treatments to be more effective than pure pharmacological treatments (Huhn et al. 2014). In addition, our goal was to identify potential moderators of the effect sizes. The meta-analysis was conducted according to the recommendations of the PRISMA Statement (Moher et al. 2009).

## Methods

### Eligibility Criteria

Studies were considered for inclusion if they (1) employed pharmacological, or combined treatments (e.g., pharmacological and psychological treatments applied at the same time); (2) used within-group, randomized, or quasi-randomized controlled study designs including a placebo intervention; (3) measured at least one of the outcome variables (i.e., global severity, frequency or financial loss); and (4) reported sufficient statistical data for effect size calculations. Studies were excluded if (1) the study was a single case study; (2) disordered gambling was secondary to Parkinson`s disease or to other medical conditions; (3) the study sample overlapped completely with the sample of another study included in the meta-analysis, or (4) no abstract or full text of the study was available.

### Information Sources and Literature Search

We conducted a multilevel literature search using the databases PsycINFO, Medline, PubMed, Psyndex, the Cochrane Central Register of Clinical Trials, ProQuest Digital Dissertations, and the web search engine Google Scholar. The search covered all relevant publications from the first available year until April 30, 2018 using the following disorder-related search terms: “pathological gambling OR gambl* OR ludomania” combined with the intervention-related key words treatment “open-label OR placebo-controlled OR random* OR trial OR pilot”. Subsequently, we conducted a thorough examination of the reference lists of review articles, meta-analyses, and original studies retrieved from the databases. Additionally, authors of relevant articles were contacted to ask for unpublished papers suitable for inclusion in the meta-analysis.

### Outcome Measures

Following the recommendations of the Banff, Alberta Consensus (Walker et al. 2006), we specified three outcome variables to measure the reduction of disordered gambling: (a) the global severity of gambling pathology, quantified by the use of valid and reliable instruments such as the Yale-Brown Obsessive–Compulsive Scale adopted for disordered gambling (PG-YBOCS; Pallanti et al. 2005), the Gambling Symptom Assessment Scale (G-SAS; Kim et al. 2009), or South Oaks Gambling Screen (SOGS; Lesieur and Blume 1987) in order to facilitate the comparability of the effect sizes; if none of these measurement tools were available for this outcome variable, we used the score for the global gambling symptomatology of the Clinical Global Impression Scale (CGI; Guy 1976); (b) frequency of gambling (e.g., number of days or hours gambled last week or month), and (c) financial loss from gambling (e.g., money wagered last week or last month), both (b) and (c) quantified using a timeline follow-up interview (Sobell and Sobell 1992), or other self-reporting forms.

### Study Selection

Study selection was performed by two independent reviewers (the first and the second authors, MG and ML), and supervised by the last author of this paper (AL). Disagreements between the authors were resolved through discussion.

### Data Collection Process and Data Extraction

We generated a structured data extraction form that we refined and modified after pilot testing a sample of 10 studies. To calculate pre-post and pre-follow-up within-group effect sizes, numerical data were extracted for each outcome separately. If different pharmacological treatments were examined within one study, data for each condition was extracted separately and treated as single within-groups for statistical analyses. To calculate controlled effect sizes, posttreatment data from placebo control groups were included. Additionally, we extracted numerical and categorical data from each study in order to perform moderator analyses. Data extraction was performed by the first author (MG), and validated by the second author (ML). Disagreements were resolved by discussion.

### Risk of Bias in Individual Studies

We assessed the internal validity of each study using the Quality Assessment Tool for Quantitative Studies, developed by the Effective Public Health Practice Project (EPHPP) (Thomas et al. 2004). This tool has demonstrated content and construct validity (Thomas et al. 2004) and is recommended for systematic reviews and meta-analyses (Deeks et al. 2003). Each study was rated in a standardized manner on six domains: selection bias, study design, identification and control of confounders, blinding, reliability and validity of data collection tools, and reporting and percentage of withdrawals and dropouts. Each domain was evaluated as strong, moderate, or weak. The global rating was calculated after evaluation of the six domains. The first two authors (MG and ML) independently assessed each study and determined the global score of each trial. Interrater reliability was quantified using the kappa statistic. Disagreements between the authors were resolved through discussion until consensus was reached.

### Effect Size Calculation and Quantitative Data Synthesis

Statistical analysis was performed using the software program Comprehensive Meta-Analysis (CMA) version 2.2.064 (Borenstein et al. 2005). We calculated the effect sizes for the reduction of global severity, frequency, and financial loss separately for within-group and controlled study designs (see Appendix for formulas). Due to small sample sizes, the effect sizes were corrected for bias using Hedges’s *g* with the corresponding 95% confidence interval (Hedges and Olkin 1984). If means and standard deviations were not available, effect sizes were calculated based on equivalent estimation procedures (e.g., *t* values, or exact probability levels). If an outcome variable was measured by more than one instrument, data from these instruments were entered separately and pooled together for the particular outcome variable (Lipsey and Wilson 2000). For studies reporting data based on both completers and ITT analyses, the ITT data was taken into account. The direction of the effect was adjusted according to the “success”: the effect size was positive if the treated group performed superior to the control group. According to Cohen’s recommendations (1977), effect sizes of 0.20–0.30 can be classified as small, those near 0.50 as medium, and those above 0.80 as large.

Assuming heterogeneity among the studies, we decided to use the random effects model for the integration of effect sizes. Heterogeneity of the effect sizes was investigated using the Q statistic with the corresponding *p* value, and the I^2^ statistic, indicating to what extent real differences in effect sizes was reflected by the proportion of the variance (Borenstein et al. 2009; Higgins et al. 2003). I^2^ values of 25%, 50%, and 75% were classified as low, moderate, and high, respectively (Higgins et al. 2003).

### Risk of Bias Across Studies

To control for publication bias, we conducted a thorough literature search and computed Rosenthal’s fail-safe *N* (Rosenthal 1979) and also examined funnel plots (Duval and Tweedie 2000). According to Rosenthal (1991), effect sizes are considered robust if the number of studies needed to obtain a nonsignificant overall effect is greater than 5*k* + 10, where *k* represents the number of studies. Additionally, we used the trim-and-fill method (Duval and Tweedie 2000) to estimate missing studies and their impact on the ascertained effect sizes. This method is based on the logic of the funnel plot and assumes a symmetrical distribution of the effect sizes for outcome variables in the absence of publication bias. In the case of asymmetrical distribution, the trim-and-fill method adjusts and corrects the effect sizes (Borenstein et al. 2009); we only applied this method if 10 studies were available for the analysis (Sterne et al. 2011). Funnel plot asymmetry was assessed by using Egger’s test (Egger et al. 1997). As singular extreme effect size values produce misleading interpretations of treatment effects (Lipsey and Wilson 2000), we used the “one-study-removed” method offered by CMA to examine the impact of each study’s effect size on the overall effect (Borenstein et al. 2005). If the recalculated results did not substantially impact the effect size and remained within the 95% CI, studies were retained in the analyses.

### Moderator Analysis

To explain heterogeneity among effect sizes, we determined the following categorical moderators: type of treatment (pharmacological vs. combined), dosage regimen (fixed vs. flexible), data analysis (intention-to-treat [ITT] vs. completer analysis), placebo lead-in phase (none vs. 1 week vs. > 1 week), type of gambling (electronic gambling vs. other types of gambling), quality of studies (EPHPP global scores), and the class of medication. The class of medication was analyzed by dividing pharmacological treatments into the following subcategories: (a) antidepressants (i.e., serotonin reuptake inhibitors [SSRI] including escitalopram/citalopram, fluvoxamine, sertraline, and paroxetine; norepinephrine-dopamine reuptake inhibitors [NDRI; e.g., bupropion]; serotonin-norepinephrine-dopamine reuptake inhibitors [SNDRI; e.g., nefazodone], and other antidepressants (e.g., agomelatine), (b) opioid-antagonists (e.g., nalmefene, naltrexone), (c) medications with mood-stabilizing potentials (e.g., lithium, topiramate, valproate, carbamazepine, olanzapine), and (d) other medications (e.g., acamprosate, *N*-acetylcysteine, memantine, tolcapone, ecopipam). Because mood disorders and anxiety were found to be associated with disordered gambling (e.g., Petry et al. 2005), and gender may influence treatment effects (Black et al. 2007a; Kim et al. 2001, 2002), we examined whether the effect sizes varied as a function of these moderators (inclusion vs. exclusion of mood disorders and/or anxiety, ≤ 50% males vs. > 50% males). Moderator analyses for categorical variables were conducted using the mixed effects model with pooled estimates of *T*^2^ and the Q-test based on analysis of variance with the corresponding *p* value for the interpretation of the differences between subgroups (Borenstein et al. 2009). In the case of at least 10 available studies (Deeks et al. 2011), we further conducted meta-regression analyses using the year of publication and the duration of treatment (assessed with the number of weeks). Meta-regression analyses on the mean age were not performed because the age across studies differs from that within studies (Thompson and Higgins 2002).

## Results

### Study Selection

**Fig. 1:**
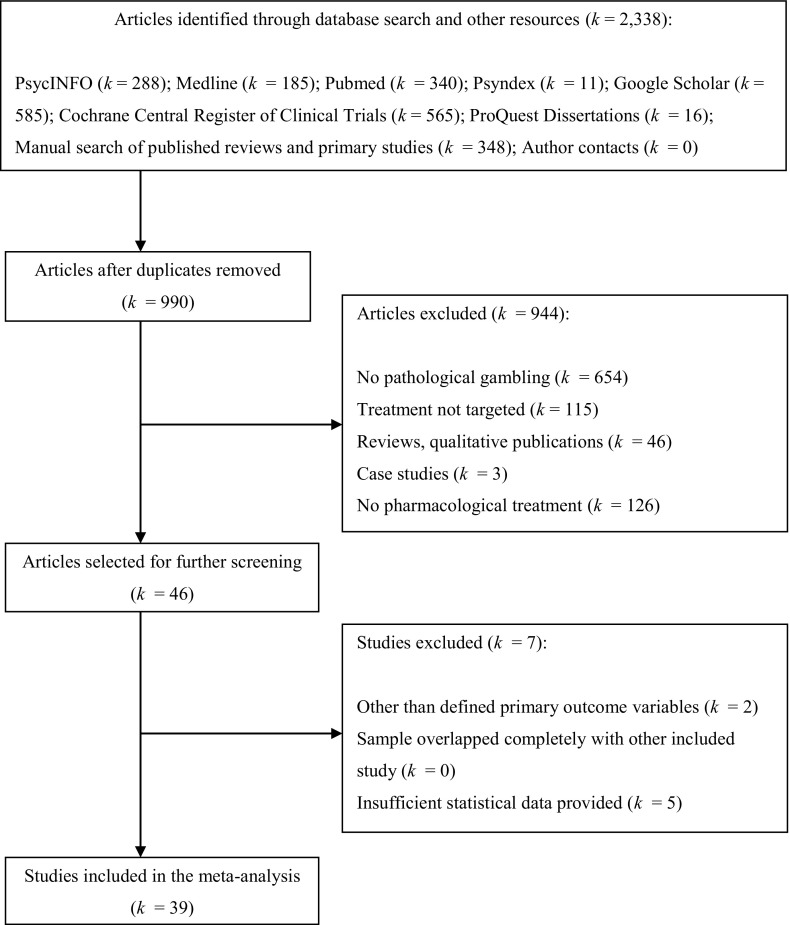
Flow diagram of the study selection process

A total of 39 studies including 43 treatment conditions were identified for inclusion in this meta-analysis. We excluded two studies assessed in recent reviews (Lupi et al. 2014; Pettorruso et al. 2014), because measurements were limited to the reduction of craving for gambling using VAS scores (Dannon et al. 2011; Zack and Poulos 2009), and thus the type of measurement tool did not satisfy the defined selection criteria. The flow diagram of the study selection process is illustrated in Fig. 1.

### Characteristics of Studies, Interventions, and Participants

The present sample of studies varied in type of control condition: Studies implemented placebo control groups (49%), no control groups (43%), or other active treatment comparisons (8%). The minority of the studies implemented a placebo lead-in phase (26%), and provided follow-up data (10%) with periods ranging from 1 week to 12 months. Results were mainly based on completers (56%).

Most trials examined antidepressants (44%), followed by opioid-antagonists (21%), mood stabilizers (21%), and other medications (14%) using a flexible dosage regimen (90%). Treatment duration ranged from 3 to 24 weeks (*M* = 11.69, *SD* = 4.59).

A total of 1340 participants across all studies were analyzed. Of those, 864 were assigned to treatment conditions, 476 individuals to control groups. Because the majority of the studies excluded participants with severe Axis I/II disorders (94%), the average levels of co-occurring mood disorders and anxiety of the participants were subclinical. The total sample was predominantly male (66%) with an average age of 43 years. Although less than half of the studies indicated the type of gambling (39%), electronic gambling was the primary activity (73%). Detailed information regarding the characteristics of studies is presented in Table 1.

**Table 1:** Characteristics of studies

References	Total *N*^a^	Treatment group(s)(*N*)/average dose/dosage regimen	Control group (*N*)	PLA lead-in (w)	Duration (w)
*Antidepressants*					
Black (2004)	10	Bupropion (10) 100–300 mg/d/flexible	None	0	8
Black et al. (2007a)	39	Burpopion (18) 324 mg/d/flexible	PLA (21)	0	12
Black et al. (2007b)	19	Escitalopram (19) 10–30 mg/d/flexible	None	0	10
Blanco et al. (2002)	32	Fluvoxamine (15) 100 mg/d (2 weeks) 200 mg/d (rest of trial)/fixed	PLA (17)	0	24
Dannon et al. (2005a)	20	(1) Topiramate (12) 200 mg/d/fixed	(2) Fluvoxamine (8)^c^ 200 mg/d/fixed	0	12
Dannon et al. (2005b)	12	Bupropion (12) 300–450 mg/d/flexible	None	0	12
Dannon et al. (2005c)	25	(1) Bupropion (12)^c^ 300–450 mg/d/flexible	(2) Naltrexone (13) 100–150 mg/d/flexible	0	12
Egorov (2017)	20	Agomelatine (20) 25–50 mg/d/flexible	None	0	8
Grant et al. (2003)	71	Paroxetine (34) 10–60 mg/d/flexible	PLA (37)	1	16
Grant and Potenza (2006)	13	Escitalopram (13) 10–30 mg/d/flexible	None	1	11
Hollander et al. (1998)	10	Fluvoxamine (10) 220 mg/d/flexible	None	8	8
Hollander et al. (2000)	10	Fluvoxamine (4) 195 mg/d/flexible	PLA (6)	1	16
Kim et al. (2002)	45	Paroxetine (23) 51.7 mg/d/flexible	PLA (22)	1	8
Myrseth et al. (2011)	15	(1) Escitalopram (15)^d^ 5–10 mg/d/flexible for 8 w, followed by (2) Escitalopram + 8 sessions CBT for 8 w (3) CBT + MI (15)^e^	None	0	8
Pallanti et al. (2002a)	12	Nefazodone (12) 345.83 mg/d/flexible	None	0	8
Ravindran and Telner (2006)	19	(1) Paroxetine (5) 10–60 mg/d/flexible (2) Paroxetine 10–60 mg/d/flexible + 12 sessions CBT (7)	PLA + 12 sessions CBT (7)^f^	0	16
Saiz-Ruiz et al. (2005)	60	Sertraline (31) 95 mg/d/flexible	PLA (29)	0	24
Zimmerman et al. (2002)	15	Citalopram (15) 34.7 mg/d/flexible	None	0	12
*Opioid antagonists*					
Dannon et al. (2005c)	25	(1) Bupropion (12) 300–450 mg/d/flexible	(2) Naltrexone (13)^g^ 100–150 mg/d/flexible	0	12
Grant et al. (2006)	73	(1) Nalmefene 25 mg/d (40) (2) Nalmefene 50 mg/d (29)^h^ (3) Nalmefene 100 mg/d (33)/fixed	PLA (44)	0	16
Grant et al. (2008a, b)	77	Naltrexone 50, 100, 150 mg/^i^ (58)/fixed	PLA (19)	1	17
Grant et al. (2010b)	128	Nalmefene 20 mg Nalmefene 40 mg (57)^h^/fixed	PLA (71)	1	3
Kim and Grant (2001)	17	Naltrexone (17) 157 mg/d/flexible	None	0	6
Kim et al. (2001)	45	Naltrexone (20) 187.5 mg/d/flexible	PLA (25)	1	11
Kovanen et al. (2016)	101	Naltrexone/flexible 50 mg in case of craving +3 sessions psychosocial support (50)	PLA + 3 sessions psychosocial support (51)	0	20
Lahti et al. (2010)	39	Naltrexone/flexible 50 mg in case of craving + 1 MI + booklet (39)	None	0	16
Toneatto et al. (2009)	52	Naltrexone 100 mg/d/flexible + 7 sessions CBT (27)	PLA + 7 sessions CBT (25)	0	10
*Mood stabilizers*					
Berlin et al. (2013)	42	Topiramate (20) 222.5 mg/d/flexible	PLA (22)	0	14
Black et al. (2008)	8	Carbamazepine (8) 675 mg/d/flexible	None	0	10
Dannon et al. (2005a)	20	(1) Topiramate (12)^k^ 200 mg/d/fixed	(2) Fluvoxamine (8) 200 mg/d/fixed	0	12
De Brito et al. (2017)	30	Topiramate 180.7 mg/d/flexible +4 sessions CR (15)	PLA + 4 sessions CR (15)	0	12
Fong et al. (2008)	21	Olanzapine (9) 2.5–10 mg/d/fixed	PLA (12)	0	7
Hollander et al. (2005a)	29	Lithium (12) 1.150 mg/d/flexible	PLA (17)	0	10
McElroy et al. (2008)	42	Olanzapine (21) 2.5–15 mg/d/flexible	PLA (21)	1	12
Pallanti et al. (2002b)	42	(1) Lithium (23) 795.6 mg/d/flexible	(2) Valproate (19) 873.7 mg/d/flexible	0	14
*Other medications*					
Black et al. (2011)	19	Acamprosate (19) 1.998 mg/d/fixed	None	0	8
Grant et al. (2007)	27	*N*-Acetylcystein (27) 600–1.800 mg/d/flexible	None	0	8
Grant et al. (2010a)	29	Memantine (29) 10–30 mg/d/flexible	None	0	10
Grant et al. (2013)	22	Tolcapone (22) 100–300 mg/d/flexible	None	0	8
Grant et al. (2014a)	22	Ecopipam/flexible (22) 50 mg in case of craving	None	1	6
Grant et al. (2014b)	28	N-Acetylcystein 1.200–3.000 mg/d/ flexible +AART + ID + MI (13)	PLA + AART + ID +MI (15)	0	12

References	FU (m)	Outcomes (assessment)	Gambling type/MD/A (+/−)^b^/% Males	Data analysis	EPHPP
*Antidepressants*					
Black (2004)	None	GS (PG-YBOCS)	NA/− ≤ 50%	CO	3
Black et al. (2007a)	None	GS (PG-YBOCS; G-SAS) FR (min/w) FL (money/w)	NA/− > 50%	ITT	3
Black et al. (2007b)	None	GS (PG-YBOCS) FR (min/w) FL (money/w)	NA/− > 50%	CO	3
Blanco et al. (2002)	None	FR (h/w) FL (money/w)	NA/− > 50%	ITT	3
Dannon et al. (2005a)	None	GS (PG-YBOCS)	NA/− > 50%	CO	2
Dannon et al. (2005b)	None	GS (PG-CGI)	NA/− > 50%	CO	3
Dannon et al. (2005c)	None	GS (PG-YBOCS)	NA/− > 50%	CO	2
Egorov (2017)	None	GS (PG-YBOCS) FR (h/w) FL (money/w)	NA/+ > 50%	CO	3
Grant et al. (2003)	None	GS (PG-YBOCS; G-SAS)	NA/− > 50%	ITT	2
Grant and Potenza (2006)	None	GS (PG-YBOCS; G-SAS)	E/+ > 50%	ITT	3
Hollander et al. (1998)	None	GS (PG-YBOCS)	Other/+ > 50%	CO	3
Hollander et al. (2000)	None	GS (PG-YBOCS)	Other/− > 50%	CO	2
Kim et al. (2002)	None	GS (G-SAS)	E/− ≤ 50%	ITT	2
Myrseth et al. (2011)	6	GS (G-SAS) FL (money/w)	E/− > 50%	ITT	3
Pallanti et al. (2002a)	None	GS (PG-YBOCS) FR (episodes/w; min./episodes) FL (money/w)	E/− > 50%	CO	3
Ravindran and Telner (2006)	None	GS (PG-YBOCS; G-SAS)	NA/− > 50%	CO	3
Saiz-Ruiz et al. (2005)	None	GS (SOGS) FR (gambling activity/w) FL (money/w)	NA/− > 50%	ITT	2
Zimmerman et al. (2002)	None	GS (PG-YBOCS) FR (days/w) FL (money/w)	E/+ > 50%	CO	3
*Opioid antagonists*					
Dannon et al. (2005c)	None	GS (PG-YBOCS)	NA/− > 50%	CO	2
Grant et al. (2006)	None	GS (PG-YBOCS; G-SAS)	NA/− > 50%	CO	3
Grant et al. (2008a, b)	None	GS (PG-YBOCS; G-SAS)	E/− ≤ 50%	ITT	2
Grant et al. (2010b)	None	GS (PG-YBOCS; G-SAS^j^)	NA/− > 50%	CO	3
Kim and Grant (2001)	None	GS (G-SAS) FR (episodes/w) FL (money/w)	NA/− ≤ 50%	ITT	3
Kim et al. (2001)	None	GS (G-SAS) FR (episodes/w)	NA/− ≤ 50%	CO	2
Kovanen et al. (2016)	None	GS (PG-YBOCS) FR (days/w) FL (highest daily expenditure/w)	E/− > 50%	ITT	2
Lahti et al. (2010)	None	GS (PG-YBOCS)	NA/− > 50%	ITT	3
Toneatto et al. (2009)	12	FR (gambling activities/w) FL (money/w)	Other/− > 50%	ITT	2
*Mood stabilizers*					
Berlin et al. (2013)	None	GS (PG-YBOCS; G-SAS)	NA/− ≤ 50%	ITT	2
Black et al. (2008)	None	GS (PG-YBOCS; G-SAS) FR (min/w) FL (money/w)	NA/− > 50%	CO	3
Dannon et al. (2005a)	None	GS (PG-YBOCS)	NA/− > 50%	CO	2
De Brito et al. (2017)	None	GS (PG-YBOCS; G-SAS) FR (h/m) FL (money/m)	E/− > 50%	CO	2
Fong et al. (2008)	None	GS (PG-CGI) FR (days/w) FL (money/d)	E/− > 50%	CO	2
Hollander et al. (2005a)	None	GS (PG-YBOCS) FR (episodes/w; min/episodes) FL (money/w)	NA/+ > 50%	CO	1
McElroy et al. (2008)	None	GS (PG-YBOCS) FR (episodes/w; h/w)	NA/− ≤ 50%	ITT	2
Pallanti et al. (2002b)	None	GS (PG-YBOCS)	E/− > 50%	ITT	2
*Other medications*					
Black et al. (2011)	None	GS (PG-YBOCS; G-SAS) FR (min/w; episodes/w) FL (money/w)	NA/− ≤ 50%	CO	3
Grant et al. (2007)	None	GS (PG-YBOCS; G-SAS) FL (money/w)	E/− > 50%	ITT	3
Grant et al. (2010a)	None	GS (PG-YBOCS; G-SAS) FR (h/w) FL (money/w)	E/− ≤ 50%	ITT	3
Grant et al. (2013)	None	GS (PG-YBOCS; G-SAS)	NA/− ≤ 50%	CO	3
Grant et al. (2014a)	None	GS (PG-YBOCS; G-SAS)	NA/− > 50%	CO	3
Grant et al. (2014b)	3	GS (PG-YBOCS)	NA/− > 50%	ITT	2

*A* anxiety, *AART* ask-advise-refer-therapy, *BDI* beck depression inventory, *CBT* cognitive behavioral therapy, *CO* completers only, *CR* cognitive restructuring, *d* day, *E* electronic gambling, *EPHPP* effective public health practice project (quality assessment tool for quantitative studies), *FL* financial loss, *FR* frequency, *FU* follow-up, *GAS* global assessment scale, *GS* global severity, *G*-*SAS* gambling symptom assessment scale, *h* hours, *ID* imaginal desensitization, *ITT* intention-to-treat, *m* month, *MD* mood disorders, *MI* motivational interviewing, *min* minutes, *NA* not available, *PG*-*CGI* clinical global impression for pathological gambling, *PG*-*YBOCS* pathological gambling adaptation of the yale-brown obsessive–compulsive scale, *PLA* placebo, *SOGS* south oaks gambling screen, *SUPP* additional support, *w* week

^a^Number of subjects included in the analysis

^b^Studies which included (+) or excluded (−) participants with comorbid MD and/or A. MD and/or A was determined based on the mean values for MD and/or A using the cut-off scores of the respective measurement tools

^c^The fluvoxamine and bupropion treatment arms were used for effect size calculations of antidepressants

^d^Only the first treatment condition was used for effect size calculations, because participants in the escitalopram and the escitalopram + CBT treatment conditions overlapped completely

^e^This treatment condition was excluded from the analyses due to the incompatibility with the selection criteria

^f^This control group was used as the comparison condition to calculate the controlled effect size for the combined treatment

^g^The naltrexone treatment arms was used for effect size calculations of opioid antagonists

^h^To ensure the comparability of nalmefene groups, data from 40 mg or 50 mg treatment arms were used for effect size calculations

^i^Only data for the combined treatment arms were reported

^j^The G-SAS scale could not be used for effect size calculation due to insufficient statistical data

^k^The topiramate treatment arm was used for effect size calculations of mood stabilizers

### Risk of Bias Within Studies

The global EPHPP scores for the studies are outlined in the Table 1. Validity assessment was conducted by two independent raters yielding an interrater reliability of κ = 0.84.

### Synthesis of Results and Risk of Bias Across Studies

**Fig. 2:**
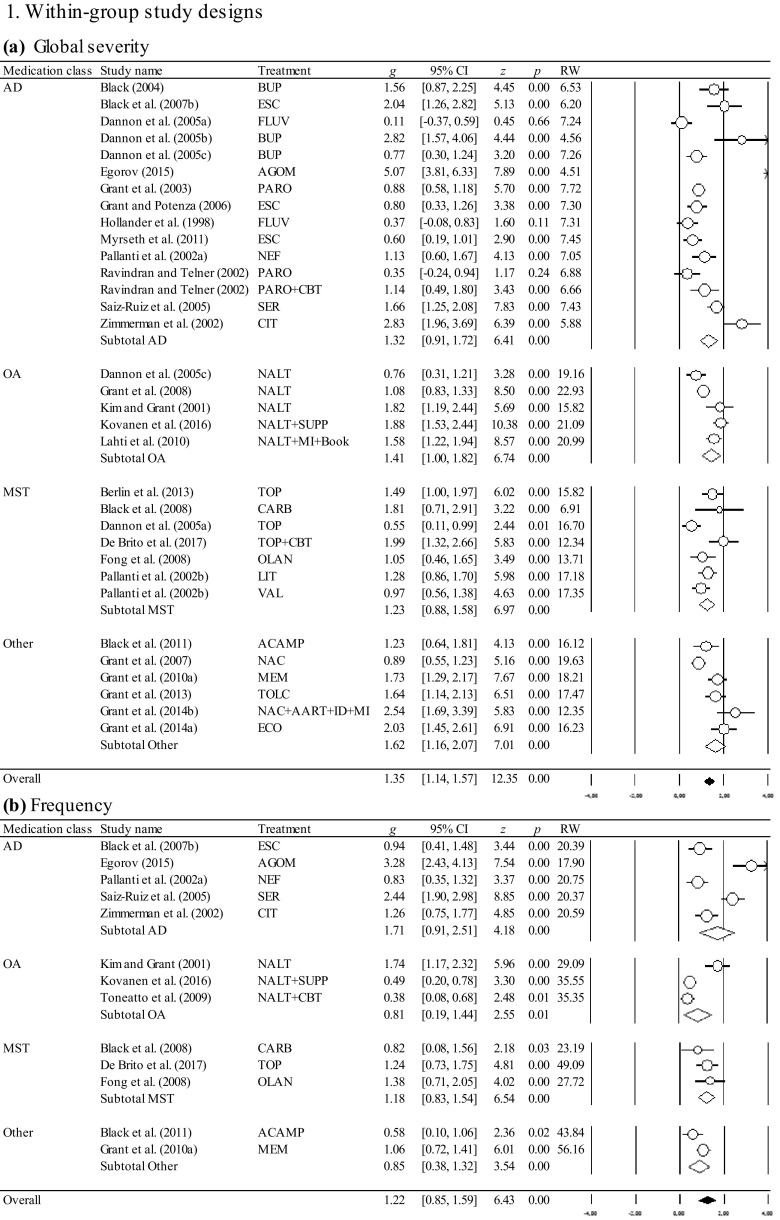

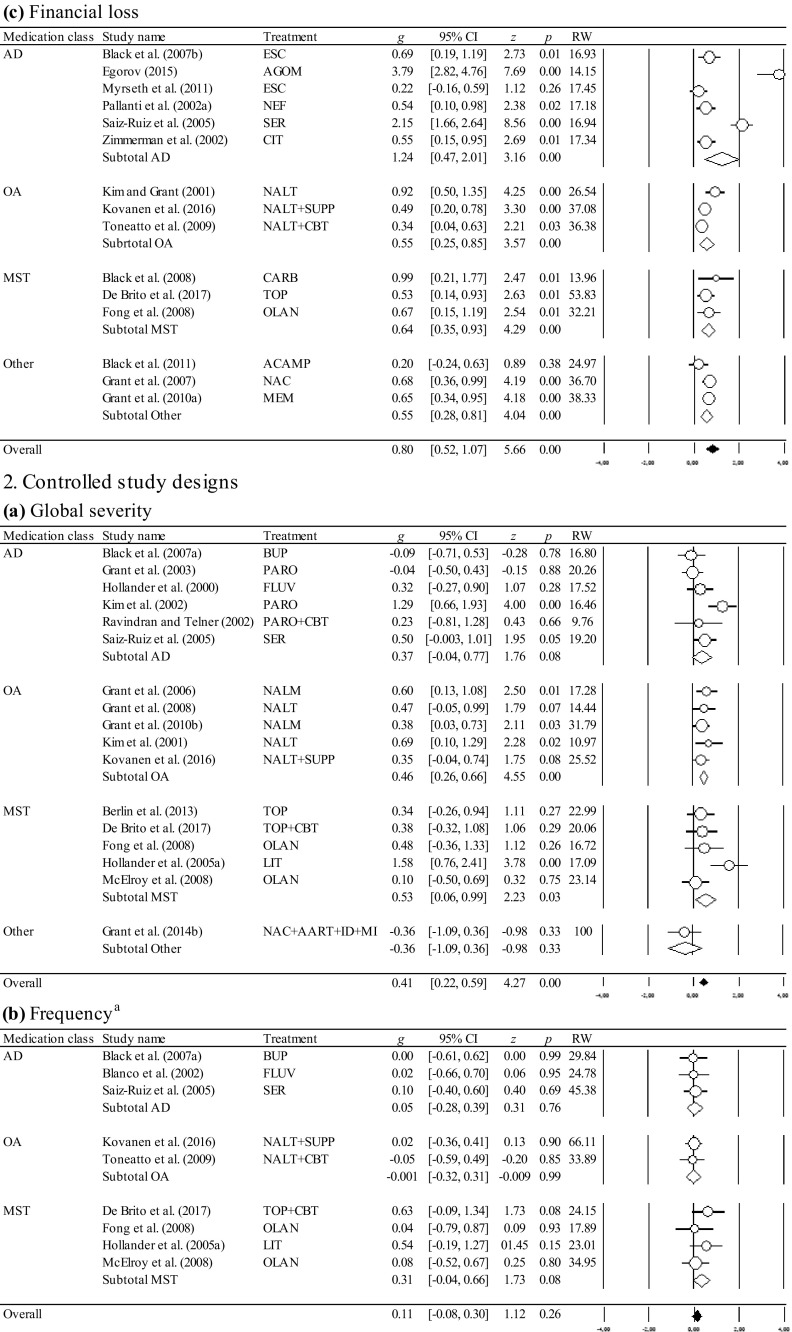

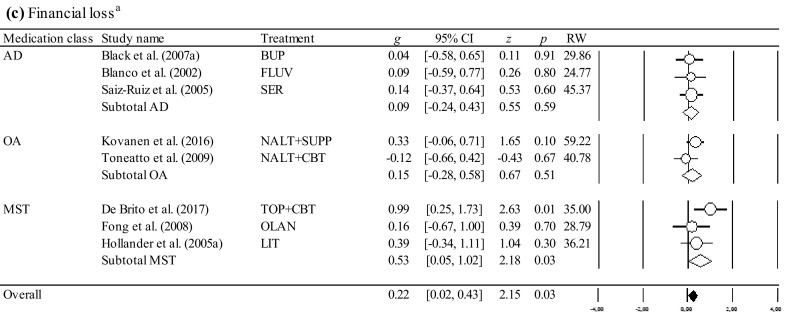
Individual and overal effect sizes for each study design, medication class and outcome at posttreatment *AART* ask-advise-refer-therapy, *ACAMP* acamprosate, *AD* antidepressants, *AGOM* agomelatine, *BUP* bupropion, *CARB* carbamazepine, *CBT* cognitive-behavioral therapy, *CI* confidence interval, *CIT* citalopram, *ECO* ecopipam, *ESC* escitalopram, *FLUV* fluvoxamine, *g* Hedges`s *g*; *ID* imaginal desensitization, *LIT* lithium, *MEM* memantine, *MI* motivational interviewing, *MST* mood stabilizers, *NAC* N-acetylcysteine, *NALM* nalmefene, *NALT* naltrexone, *NEF* nefazodone, *OA* opioid antagonists, *OLAN* olanzapine, *PARO* paroxetine, *RW* relative weight, *SER* sertraline, *SUPP* psychosocial support, *TOLC* tolcapone, *TOP* topiramate, *VAL* valproate. ^a^Data for “other medications” were not available

The overall and the medication class-specific within-group and controlled effect sizes on all outcomes at posttreatment and follow-up, the 95% CI, and the significance tests are shown in Table 2. Results of the within-group and controlled effect sizes and their forest plots are presented in Fig. 2.

**Table 2:** Effect sizes for pharmacological treatments at posttreatment and follow-up

Outcome	Effect	Within-group study designs							Controlled study designs						
		*k*	*g*	95% CI	*z*	*p*	*I* ^2^	FS *N*	*k*	*g*	95% CI	*z*	*p*	*I* ^2^	FS *N*
Overall															
GS	Post	33	1.35	[1.14; 1.57]	12.35	< 0.001	83.96***	7166	17	0.41	[0.22; 0.59]	4.27	< 0.001	44.48*	131
	FU	3	1.63	[0.81; 2.44]	3.92	< 0.001	83.64**	65	1	1.31	[0.35; 1.91]	2.84	< 0.01	0.00	–^b^
FR	Post	13	1.22	[0.85; 1.59]	6.43	< 0.001	87.53***	899	9	0.11	[− 0.08; 0.30]	1.12	0.264	0.00	–^a^
	FU	1	0.47	[0.11; 0.82]	2.59	< 0.05	0.00	–^b^	1	− 0.03	[− 0.65; 0.59]	− 0.10	0.924	0.00	–^b^
FL	Post	15	0.80	[0.52; 1.07]	5.66	< 0.001	85.13***	685	8	0.22	[0.02; 0.43]	2.15	< 0.05	0.00	2
	FU	2	0.27	[0.01; 0.52]	2.07	< 0.05	0.00	–^b^	1	− 0.18	[− 0.81; 0.44]	− 0.58	0.565	0.00	–^b^
AD															
GS	Post	15	1.32	[0.91; 1.72]	6.41	< 0.001	87.94***	988	6	0.37	[− 0.04; 0.77]	1.76	0.078	63.08*	–^a^
FR	Post	5	1.71	[0.91; 2.51]	4.18	< 0.001	90.14***	200	3	0.05	[− 0.28; 0.39]	0.31	0.759	0.00	–^a^
FL	Post	6	1.24	[0.47; 2.01]	3.16	< 0.01	93.57***	159	3	0.09	[− 0.24; 0.43]	0.55	0.586	0.00	–^a^
OA															
GS	Post	5	1.41	[1.00; 1.82]	6.74	< 0.001	82.70***	341	5	0.46	[0.26; 0.66]	4.55	< 0.001	0.00	24
FR	Post	3	0.81	[0.19; 1.44]	2.55	< 0.05	88.82***	33	2	− 0.001	[− 0.32; 0.31]	− 0.009	0.993	0.00	–^b^
FL	Post	3	0.55	[0.25; 0.85]	3.57	< 0.001	59.53	22	2	0.15	[− 0.28; 0.58]	0.67	0.505	41.79	–^b^
MST															
GS	Post	7	1.23	[0.88; 1.58]	6.97	< 0.001	65.87**	254	5	0.53	[0.06; 0.99]	2.23	< 0.05	54.35	10
FR	Post	3	1.18	[0.83; 1.54]	6.54	< 0.001	0.00	29	4	0.31	[− 0.04; 0.66]	1.73	0.084	0.00	–^a^
FL	Post	3	0.64	[0.35; 0.93]	4.29	< 0.001	0.00	13	3	0.53	[0.05; 1.02]	2.18	< 0.05	16.40	2
Other															
GS	Post	6	1.62	[1.16; 2.07]	7.01	< 0.001	78.56***	336	1	− 0.36	[− 1.09; 0.36]	− 0.98	0.327	0.00	–^b^
FR	Post	2	0.85	[0.38; 1.32]	3.54	< 0.001	60.98	–^b^	NA						
FL	Post	3	0.55	[0.28; 0.81]	4.04	< 0.001	43.08	20	NA						

*AD* antidepressants, *CI* confidence interval, *FR* frequency, *FL* financial loss, *FS N* fail-safe *N* (number of studies needed to obtain a nonsignificant treatment effect), *FU* effect sizes from pretreatment to latest follow-up for within-group study designs, and from posttreatment to latest follow-up for controlled study designs, *g* Hedges’s *g*, *I*^2^ percentage of total variation across studies, *GS* global severity, *k* number of treatment conditions, *MST* mood stabilizers, *NA* not available, *OA* opioid antagonists, *Q*_*bet*_ homogeneity statistic for differences between subgroups

^a^Fail-safe *N* was not calculated because *p* was not significant

^b^Fail-safe *N* was not calculated because less than three studies were available

*p < 0.05

**p < 0.01

***p < 0.001

### Effect Sizes of Within-Group Study Designs at Posttreatment and Follow-Up

At posttreatment, the overall and medication-class-specific effect sizes were significant ranging from medium to large for the outcome variables. At follow-up, the overall analyses revealed significant results with a large effect size for the reduction of global severity. Follow-up data for the remaining outcome variables was based on single trials precluding the interpretation of long-term effect sizes. As depicted in Table 2, I^2^ values suggested predominantly high variability across the studies beyond sampling error. The trim-and-fill method identified 7 studies causing funnel plot asymmetry for the reduction of global severity (Egger’s test, *p* < 0.01), and 5 studies for the reduction of frequency (Egger’s test, *p* < 0.01). Accounting for the asymmetry of the funnel plot by filling in studies suggested a slightly reduced effect size for the reduction of global severity (*g* = 1.11; 95% CI [0.87, 1.34]), and a reduced effect size for the reduction of frequency (*g* = 0.74; 95% CI [0.33, 1.15]), suggesting small publication bias. No indication for publication bias was found for the reduction of financial loss (Egger’s test *p* = 0.045). The fail-safe *N* analyses performed for the available data suggested the robustness of the effect sizes, except for the reduction of financial loss among opioid antagonists, mood stabilizers and other medications which were not robust.

Although one study (Egorov 2017) produced very large effect sizes in terms of all outcome variables (see Fig. 2), outlier identification through the one-study-removed procedure showed no impact of any single study on the overall effects or on the between-study heterogeneity.

### Effect Sizes of Controlled Study Designs at Posttreatment and Follow-Up

At posttreatment, the overall and medication-class-specific effect sizes ranged from negative to medium. Only the overall effect sizes were significant and medium for the reduction of global severity, and small for the reduction of financial loss. Among the medication classes, opioid antagonists and mood stabilizers produced significant and medium effect sizes for the reduction of global severity. For mood stabilizers, a significant and medium effect size was also observed for the reduction of financial loss. Follow-up data was based on single trials precluding the interpretation of long-term effect sizes. I^2^ values suggested predominantly low variability across the studies beyond sampling error. No indication for publication bias was found for the reduction of global severity (Egger’s test *p* = 0.288). The fail-safe *N* analyses performed for the available data suggested the robustness only for the overall effect size for the reduction of global severity. The remaining effect sizes were not robust.

### Moderator Analyses

Moderator analyses were conducted on the overall and medication-specific effect sizes. The results of categorical variables are presented in the Table 3.

**Table 3:** Moderator analyses for categorical variables for pharmacological treatments

Moderator	Outcome variable	Within-group study designs		Controlled study designs	
		*Qbet*	*p(Q)*	*Qbet*	*p(Q)*
Treatment (pharmacological, combined)	GS	3.34	0.067	1.39	0.239
	FR	3.35	0.067	0.00	0.938
	FL	1.66	0.197	0.52	0.469
Medication class (AD, OA, MST, other)	GS	1.59	0.663	3.32	0.345
	FR	4.31	0.230	1.83	0.400
	FL	3.24	0.357	2.62	0.270
AD type (SSRI/SNDRI, NDRI, agomelatine)	GS	20.97	< 0.001	0.93	0.335
	FR	4.80	< 0.05	0.04	0.850
	FL	11.09	< 0.01	0.05	0.825
OA type (naltrexone, nalmefene)	GS	0.00	1.00	0.01	0.917
	FR	0.00	1.00	0.00	1.00
	FL	0.00	1.00	0.00	1.00
MST type (carbamazepine, lithium, olanzapine, topiramate, valproate)	GS	0.65	0.958	8.21	< 0.05
	FR	0.04	0.982	2.16	0.339
	FL	0.11	0.946	2.39	0.302
Other type (acamprosate, ecopipam, memantine, *N*-acetylcysteine, tolcapone)	GS	0.25	0.993	0.00	1.00
	FR	2.56	0.109	NA	NA
	FL	3.51	0.173	NA	NA
Dosage regimen (fixed, flexible)	GS	5.01	< 0.05	0.18	0.671
	FR	0.32	0.572	0.11	0.742
	FL	1.04	0.309	0.17	0.679
Data analysis^a^ (CO, ITT)	GS	0.009	0.923	1.86	0.172
	FR	0.03	0.873	1.67	0.102
	FL	0.11	0.741	2.50	0.114
Placebo lead-in (none, 1 week, 8 weeks)	GS	3.31	0.191	0.04	0.850
	FR	1.88	0.171	0.34	0.559
	FL	0.82	0.366	1.79	0.181
Type of gambling^b^ (electronic, other)	GS	3.38	0.066	0.40	0.527
	FR	0.89	0.346	0.59	0.442
	FL	1.38	0.241	1.77	0.183
EPHPP (1, 2, 3)	GS	1.99	0.158	0.25	0.619
	FR	0.09	0.764	0.22	0.642
	FL	0.00	0.972	0.61	0.434
Comorbid MD/A (Included, Excluded)	GS	1.88	0.171	6.98	< 0.01
	FR	0.53	0.468	0.06	0.811
	FL	1.68	0.195	0.66	0.415
% Males (≤ 50%, > 50%)	GS	0.46	0.498	1.15	0.284
	AD	0.09	0.759	10.18	< 0.01
	FR	0.09	0.770	0.01	0.905
	FL	0.55	0.458	0.00	1.00

Moderator analyses were conducted on the overall and medication-specific effect sizes. Only if moderator analyses on the medication-specific effect sizes differed from those on the overall effect sizes, results were reported separately

*A* anxiety, *AD* antidepressants, *CO* completers, *EPHPP* effective public health practice project (quality assessment tool for quantitative studies), *FR* frequency, *FL* financial loss, *GS* global severity, *ITT* intention-to-treat analysis, *MD* mood disorders, *MST* mood stabilizers, *NA* not available, *NDRI* norepinephrine-dopamine reuptake inhibitor, *OA* opioid antagonists, *Q*_*bet*_ homogeneity statistic for differences between subgroups, *SSRI* serotonin reuptake inhibitor, *SNDRI* serotonin-norepinephrine-dopamine reuptake inhibitor

^a^The study of Black (2004) was excluded, because no information regarding the data analysis was available

^b^Only studies which reported the type of gambling were included in the analyses

The effect sizes across both study designs were not moderated by the type of treatment, the type of data analysis, placebo lead-in phase, the type of gambling, and treatment duration. Considering within-group study designs, significantly larger effect sizes were found for studies using flexible compared to fixed dosage regimen, and for those published more recently with respect to the reduction of global severity (β = 0.06; SE = 0.02; *p* < 0.01). Within the medication classes, agomelatine showed significantly larger effect sizes compared to other antidepressants in regards of all outcome variables.

In controlled study designs, lithium showed an advantage over other mood stabilizers for the reduction of global severity. This result, however, was based on a single trial that included individuals with bipolar disorders (Hollander et al. 2005a) impacting the moderator “comorbid mood disorders/anxiety” accordingly. Similarly, one trial recruiting predominantly female participants (Kim et al. 2002) produced a larger effect size compared to those including mainly male participants among antidepressants.

## Discussion

The objective of this paper was to investigate the efficacy of pharmacological treatments for disordered gambling and to identify possible predictors of treatment outcome. Results from within-group study designs revealed that pharmacological treatments effectively reduced the global severity and financial loss from gambling at short-term. We also ascertained a strong effect size for the reduction of frequency; however, the result from the trim-and-fill analysis indicated asymmetry in the underlying study sample. Although this could be caused by publication bias, it is more reasonable to assume that funnel plot asymmetry arose from between-study heterogeneity (Egger et al. 1997; Sterne et al. 2001). Furthermore, the robustness of the treatment effect to reduce gambling frequency is supported by the fail-safe *N* analysis. Treatment success for the reduction of global severity remained stable over longer periods; however no firm conclusions can be drawn on the long-term gains of pharmacological treatments for the remaining outcome variables due to the limited amount of data. Similar levels of short-term effect sizes and the lack of catamnestic data were reported in previous meta-analyses in this area (Leibetseder et al. 2011; Pallesen et al. 2007). Direct comparisons with the present meta-analysis, however, are problematic because effect sizes were pooled across within-group and controlled study designs.

As expected, inferior results for medications were observed in controlled study designs suggesting high rates of placebo responses. A number of reasons might account for these findings. First, mediators such as the therapeutic alliance established by regular contacts between patients and therapists, patients` expectations to benefit from treatment, learning processes associated with drug stimuli (classical conditioning), elevated levels of motivation to change problematic behavior, or the natural recovery from gambling, all aspects which are extensively discussed in the literature (e.g., Finniss et al. 2010; Grant and Chamberlain 2017; Prochaska et al. 1992; Schedlowski et al. 2015; Slutske 2006), may have contributed to the small between-group differences. Alternatively, additional support (i.e., keeping diaries, participation in Gamblers Anonymous groups, or self-help programs) which was either recommended or not monitored in some trials may have influenced the treatment effects. Despite these limitations, we ascertained significant medium and small benefits of medications relative to placebo for reducing the global severity and financial loss from gambling. Except for the overall effect size for the global symptom severity, however, our results should be interpreted with caution due to the lack of robustness demonstrating the need for further research. Nevertheless, the magnitude of the effect for the global symptom severity corresponds to that found for a variety of medical diseases and mental health disorders revealing a median of all effect sizes of 0.40 (Leucht et al. 2012). As observed across both study designs, the implementation of short, one-week placebo lead-in phases did not affect treatment response. In the light of an early and high placebo effect observed for disordered gambling (Hollander et al. 1998, 2000), more extended run-in phases may lead to a more effective identification of placebo responders.

Moderator analyses indicated a small and nonsignificant advantage of combined treatments over pharmacotherapy alone for the reduction of global severity in within-group study designs and the reduction of financial loss in controlled study designs partially supporting our hypothesis. Although our findings were based on a limited number of combined trials, they agree with the tendency found in a recent review of meta-analyses on a range of medical diseases and disabling conditions (Huhn et al. 2014). With regard to the limitations of placebo controlled designs, it should be noted that particularly combined treatments examining medications along with psychotherapies which were equally provided to the placebo groups appear susceptible for masking the drug effect (Kovanen et al. 2016). Therefore, separate treatment groups receiving medication and psychotherapy alone compared with the combined treatment may help to disentangle the efficacies of the relevant treatment elements in upcoming studies (see also De Brito et al. 2017; Kovanen et al. 2016).

Improvement was independent from treatment duration and gambling type across both study designs. The latter finding agrees with that reported in meta-analyses on psychological treatments for disordered gambling (Gooding and Tarrier 2009; Goslar et al. 2017) suggesting that all gamblers may share common mechanisms of addiction which were reduced during treatment. Moreover, individuals with and without co-occurring mood disorders and/or anxiety benefited to a comparable degree from treatment underscoring the conclusions of a recent review (Dowling et al. 2016). Only lithium appeared to be most effective for gamblers with bipolar disorder (Hollander et al. 2005a) supporting the treatment algorithm for this subgroup of individuals (Bullock and Potenza 2012). Given the preliminary nature of these results, future studies should systematically investigate and report the types and rates of co-occurring disorders in order to identify subgroups of gamblers, and to determine the impact of comorbidity on treatment outcomes (Dowling et al. 2016). Similarly, we found no advantage of any medication class over the other in reducing symptoms of disordered gambling across both study designs (see also Bartley and Bloch 2013; Pallesen et al. 2007). Regarding within-group study designs, however, agomelatine outperformed SSRIs, SNDRI and NDRI. This novel antidepressant that promotes the resynchronization of circadian rhythms by acting on melatonin and 5-HT_2c_ receptors (Le Strat and Gorwood 2008) produced promising results in the treatment of mood, anxiety, and a range of other disorders (for a review see De Berardis et al. 2015). Since our findings were based on a single trial including a small number of participants (Egorov 2017), further research is required to substantiate the efficacy of agomelatine for the treatment of disordered gambling. The particularly large effect size of this study and the beneficial gains of recently published treatments on topiramate (De Brito et al. 2017), naltrexone (Kovanen et al. 2016), *N*-acetylcysteine (Grant et al. 2007; Grant et al. 2014b), and ecopipam (Grant et al. 2014a) may have caused the positive association between outcome and year of publication and the superiority of flexible over fixed dosage regimen for the reduction of global severity. Although a slight advantage for flexible dosage was found for the remaining outcome variables, results should be interpreted with caution and warrant further research.

Also in line with the current state of knowledge emphasizing opioid antagonists as the most supported drug treatment for gambling disorder (Bartley and Bloch 2013; Bullock and Potenza 2012), we ascertained a significant and medium advantage of opioid antagonists over placebo for the reduction of global severity. In contrast to Bartley and Bloch (2013), however, who found substantial heterogeneity across the studies and an effect size being flawed by the type of data analysis and the year of publication, our analyses revealed between-study homogeneity with no moderators impacting the effect size for opioid antagonists. These differences may be based on the fact that Bartley and Bloch (2013) pooled effect sizes across scales with different contents (i.e., global severity, frequency and financial loss were subsumed under the single outcome variable “gambling severity”), and across varying measurement tools. Therefore, future studies and meta-analyses are encouraged to select equivalent response measures and differentiate between distinct aspects of gambling behaviors as recommended by the Banff, Alberta Consensus (Walker et al. 2006) in order to collect more information about the impact of treatment on frequency and financial loss from gambling, and to facilitate comparisons across the studies (for a review see Pickering and Keen 2018). Besides opioid antagonists which proved effective not only for the treatment of alcohol dependence (e.g., Jonas et al. 2014), but also for behavioral addictions other than disordered gambling (for a review see Mouaffak et al. 2017), we observed significant superiority of mood stabilizers over placebo supporting our hypothesis. It should be noted, however, that these results were driven by single trials which produced strong effects including either gamblers with bipolar disorders treated with lithium (Hollander et al. 2005a), or those treated with topiramate coupled with a brief cognitive intervention (De Brito et al. 2017; see Fig. 2 for the reduction of financial loss). In addition to topiramate, other glutamatergic agents such as *N*-acetylcysteine and acamprosate which are favorable treatment options for substance use disorders (Guglielmo et al. 2015; Minarini et al. 2017; Witkiewitz et al. 2012), produced promising results in noncontrolled trials for disordered gambling (Black et al. 2011; Grant et al. 2007). Since medications targeting glutamatergic pathways not only appear to reduce symptoms of craving, but may also enhance cognitive flexibility as demonstrated by the use of memantine (Grant et al. 2010a), these types of drugs seem promising for investigation in further controlled study designs (Pettorruso et al. 2014). Relative to placebo, antidepressants reduced the global symptom severity to a similar level compared to that of the remaining drugs. The lack to obtain a significant effect, however, may be due to the heterogeneity across the studies caused by a single trial that determined the magnitude of the treatment response and differed from the others by yielding a large effect size and including a high percentage of female participants (Kim et al. 2002). Even though a variety of gender-specific differences were ascertained in clinical trials (e.g., Echeburua et al. 2011), the impact of sex on treatment outcomes needs to be replicated. Moreover, antidepressants may act differentially for subgroups of gamblers with additional diagnoses other than mood disorders and/or anxiety which were not systematically assessed. For example, SSRIs may be beneficial for gamblers with obsessive–compulsive disorders, but not for individuals with attention-deficit/hyperactivity disorder (Hollander et al. 2005b) underpinning the need to investigate comorbid conditions (Dowling et al. 2016).

Nevertheless, some limitations and implications for further research should be mentioned: First and foremost, our meta-analysis covered a small number of studies. However, the short-term within-group effect sizes, and the overall controlled effect size for the reduction of global severity were robust. Second, as is true for most meta-analytic reviews, the included studies differed in their methodological quality, although when addressed statistically, we did not observe a systematic bias in the effect sizes due to differences in the quality of the studies. It should be noted that none of the studies achieved the highest rating reflecting limited quality of evidence with respect to selection bias, high dropout rates, and—particularly regarding within-group study designs—to the identification and control of confounders, and blinding. As a result, rigorously designed, large-scale RCTs are necessary including extended placebo lead-in periods, the monitoring of additional psychosocial support, the type of comorbidity, the use of equivalent measurement tools, the reporting of outcome variables according to the Banff, Alberta Consensus (Walker et al. 2004), and the provision of follow-up data in order to determine the efficacy of medications over the long-term. Although the amount of drug dosage has demonstrated to impact treatment outcome (e.g., Bloch et al. 2010), this moderator could not be addressed in the present meta-analysis, because the studies often provided insufficient information. Furthermore, when this information was provided, the dosage often varied depending on the type of medication, complicating these analyses, which would have resulted in insufficient test power. Moreover, the dosage within each drug class usually showed little variation, further complicating these analyses (Thompson and Higgins 2002). Therefore, additional studies will be necessary to examine the impact of dosage on treatment outcome within the different drug classes. Moreover, separate data should be reported for participants who receive low, moderate or high doses of the relevant medication facilitating the comparability of dosage within each drug class.

Despite these limitations, the results of the present meta-analysis suggest that a variety of medications are effective for the management of gambling behaviors. Focusing placebo controlled designs, opioid antagonists and mood stabilizers, particularly the glutamatergic agent topiramate combined with a cognitive intervention and lithium for gamblers with bipolar disorders demonstrated preliminary evidence for reducing the global gambling severity. Although further neurobiological and neuroimaging studies should promote a better understanding in the mechanisms underlying problematic gambling behavior (e.g., Bullock and Potenza 2012), it seems most important to investigate the reasons for the high placebo response rates (Grant and Chamberlain 2017) and the natural recovery (e.g., Cuijpers and Cristea 2015) in order to improve pharmacological treatments for disordered gambling.

## Appendix

Formulas for the Effect Size Calculations

To compute the within-group effect sizes, the following formulas were utilized (Borenstein et al. 2005, 2009):

$$d = \left( {\frac{{\overline{{Y_{1} }} - \overline{{Y_{2} }} }}{{S_{Difference} }}} \right)\sqrt {2(1 - r)} ,$$

such that $\overline{Y}$_1_ reflects the pretreatment mean, $\overline{Y}$_2_ reflects the post-treatment mean, *S*_*difference*_ reflects the standard deviation of the difference, and *r* reflects the correlation between pretreatment and posttreatment scores. Following Rosenthal (1991), we estimated the pre-post correlation to be *r* = 0.70. Due to small sample sizes, all effect sizes were corrected for bias using Hedges’s *g* which was computed by multiplying *d* with the correction factor

$$J(df) = 1 - \frac{3}{4df - 1},$$

such that *df* represents the degrees of freedom to estimate the within-group standard deviation. These formulas were also applied for the calculation of effect sizes from pretreatment to the latest follow-up.

The controlled effect sizes were computed using the following formula:

$$g = \frac{{\left( {\overline{\gamma }_{1} - \overline{\gamma }_{2} } \right)}}{{\sqrt {\frac{{\left( {n_{1} - 1} \right)SD_{1}^{2} + \left( {n_{2} - 1} \right)SD_{2}^{2} }}{{n_{\text{Total}} - 2}}} }} \times \left( {1 - \frac{3}{{4\left( {n_{\text{Total}} - 9} \right)}}} \right),$$

such that $\overline{Y}$_1_ is the mean of the treatment group, $\overline{Y}$_2_ is the mean of the control group at posttreatment, *SD*_*1*_ and *SD*_*2*_ are the standard deviations of post-treatment scores of the treatment and control group, *n* is the sample size. This formula was also applied for the calculation of effect sizes from posttreatment to the latest follow-up.
